# Management of Traumatic Cardiac Arrest from hypovolaemia: is there a consensus?

**DOI:** 10.1186/1757-7241-23-S2-A11

**Published:** 2015-09-11

**Authors:** Naomi Pritchard, Rosel Tallach

**Affiliations:** 1 Department of Anaesthetics, The Royal London Hospital, Barts Health, London, UK

## Background

The survival rates of Traumatic Cardiac Arrest (TCA) from hypovolaemia remain poor. The underlying pathology differs from medical cardiac arrest and therefore necessitates different management. This has been described in the pre-hospital and resuscitation room setting.

Our hypothesis was that response to cardiac arrest from exsanguinating haemorrhage varied widely when it occurred downstream of the emergency department. We gleaned national opinion on how to manage this pathology when it occurred in transit to, or in, the operating theatre.

## Method

A telephone/email survey of all English major trauma centres (MTCs) asked two questions:

1) Did their MTC have an algorithm for in-hospital TCA differing from the ALS cardiac arrest algorithm?

2) If a patient arrested from presumed ongoing haemorrhage, would they receive cardiopulmonary resuscitation (CPR), Adrenaline or just continued filling?

## Results

14 out of 22 MTCs responded. 3 have protocols specifically for in-hospital TCA, all differing significantly.

Responses from the other 10 MTCs varied. 5 centres follow ALS medical protocols and commence CPR. 1 centre would start CPR, but stop for thoracotomy in the case of penetrating trauma. Of those centres not starting CPR, 3 undertake thorocotomy to achieve aortic compression. 2 centres decide on a case to case basis.

ALS protocol followers use 1mg Adrenaline. Some centres give ‘some’ Adrenaline, 7 centres don't give Adrenaline. 1 centre didn't know if they would or not. Nowhere favoured just filling and no other action.

## Conclusion

There is no consensus on in-hospital treatment of TCA caused by hypovolaemia.

Wide variations in staff training and experience influences TCA management nationally. While much training has been invested in emergency physicians, this training has not extended to anaesthetists who are the team leaders downstream of the emergency department. Confusion regarding the resuscitation from haemorrhage/hypovolaemia may contribute to its high mortality.

